# Resveratrol Relieved Acute Liver Damage in Ducks (*Anas platyrhynchos*) Induced by AFB1 via Modulation of Apoptosis and Nrf2 Signaling Pathways

**DOI:** 10.3390/ani11123516

**Published:** 2021-12-10

**Authors:** Fangju Liu, Yingjie Wang, Xin Zhou, Mengru Liu, Sanjun Jin, Anshan Shan, Xingjun Feng

**Affiliations:** Laboratory of Molecular Nutrition, Institute of Animal Nutrition, Northeast Agricultural University, Harbin 150030, China; liufangju0701@163.com (F.L.); 18846091206@163.com (Y.W.); zhouxinzhouxin1996@126.com (X.Z.); liumengruu@163.com (M.L.); Sanjunjin@163.com (S.J.); asshan@neau.edu.cn (A.S.)

**Keywords:** resveratrol, aflatoxin B1, acute liver injury, oxidative stress, apoptosis, ducks

## Abstract

**Simple Summary:**

Aflatoxin B1 is ubiquitous in food and feed, which not only poses a great threat to animals, but also affects human health. It is unclear whether resveratrol can resist aflatoxin B1 damage in ducks’ livers. Therefore, the effect of resveratrol supplementation in the diets on liver injury aflatoxin B1was investigated through the gavage of aflatoxin B1. It was found that a diet that includes resveratrol can effectively protect ducks’ livers from acute injury caused by aflatoxin B1. Our study suggests that resveratrol serves as a potential phytochemical feed additive for the treatment of acute aflatoxin B1 poisoning in ducks

**Abstract:**

The presence of aflatoxin B1 (AFB1) in feed is a serious threat to livestock and poultry health and to human food safety. Resveratrol (Res) is a polyphenolic compound with antioxidant, anti-apoptotic and other biological activities; however, it is not clear whether it can improve AFB1 induced hepatotoxicity. Therefore, this study was conducted to investigate the effects of dietary Res on liver injury induced by AFB1 and its mechanisms. A total of 270 one-day-old male specific pathogen free (SPF) ducks, with no significant difference in weight, were randomly assigned to three groups: the control group, the AFB1 group and the AFB1 + Res group, which were fed a basic diet, a basic diet and a basic diet containing 500 mg/kg Res, respectively. On the 70th day, the ducks in theAFB1 group and the AFB1+ 500 mg/kg Res group were given 60 μg/kg AFB1 via gavage. When comparing the AFB1 group and the AFB1 + Res group and also with the control group, AFB1 significantly increased liver damage, cytochrome P450 (CYP450) and AFB1-DNA adduct content, increased oxidative stress levels and induced liver apoptosis, which was improved by Res supplementation. In sum, the addition of Res to feed can increase the activity of the II-phase enzyme, activate the nuclear factor E2-related factor 2 (Nrf2) signal pathway, and protect ducks’ livers from the toxicity, oxidative stress and inflammatory reaction induced by AFB1.

## 1. Introduction

China has the largest number of ducks in the world. Mycotoxins are ubiquitous in food and feed, not only posing a huge threat to the meat duck breeding industry, but also posing as a major risk factor that affects human and animal health [1]. Aflatoxin is a secondary metabolite of *Aspergillus flavus* and *Aspergillus parasiticus*. Aflatoxin can be toxic to almost any animal [2], and poultry is the most sensitive to the toxicity and carcinogenicity of aflatoxin B1 (AFB1). AFB1 can reduce animal growth performance and immunity function rates, decrease the egg production of hens, increase the susceptibility of animals to disease, and have other adverse effects on animals [3,4,5,6]. The liver is the main metabolic and detoxification organ of AFB1 and is also the main target organ in AFB1 toxic attack [7]. AFB1 induces the overproduction of reactive oxygen species (ROS) and oxidative stress in the liver, which results in the cell degradation of proteins, lipids and DNA, apoptosis, and autophagy, and can further result in liver necrosis, sclerosis, acute liver damage, and even liver tumors in animals [3,8]. The metabolizing AFB1 enzymes have traditionally been divided into two groups: drug-metabolizing enzymes of phase I, which can be mediated by the micro-mitochondrial oxidase of the superfamily cytochrome P450 (CYP 450) gene [9]; and drug-metabolizing enzymes of phase II drugs that catalyze detoxification mediated by glutathione transferase (GST), such as GSTA, GSTM and GSTS [10,11,12]. AFB1 that is absorbed in the body is metabolized by phase I metabolic enzymes (mainly cytochrome P450 oxidase family members, such as CYP1A2, CYP3A4, CYP2A6, etc.) to a variety of metabolites, e.g., aflatoxin M1 (AFM l), aflatoxin Pl (AFP 1), aflatoxin Ql (AFQ 1), and aflatoxin alcohol [13]. AFM l, AFP 1 and AFQ 1 are inactive, and are excreted directly by urine or by feces after being combined with glucuronic acid via transferase catalysis, while aflatoxin alcohol continues to have a toxic effect on the liver [14]. The main compound of aflatoxin alcohol, AFB1-exo-8, 9-epoxide (AFBO), can be combined with 7th Nitrogen atom (N7) in the amino acid residues of guanosine G in the DNA chain, and forms the main adduct precursor which causes DNA mutations and severe liver damage [15]. In addition, AFBO can be detoxified by transforming epoxide hydrolase and phase II metabolic enzyme glutathione thiotransferase into AFB1-dihydrodiol and uric acid with lower toxicity [16]. However, the activation of the CYP 450 enzyme system can produce a large amount of ROS and cause oxidative stress in the liver [17].

Oxidative stress plays a key role in the toxicity mechanism of AFB1 [18]. Therefore, the addition of antioxidants to animal feed can reduce the toxicity of AFB1 to animals by enhancing their antioxidant system and immunity. In recent years, Nrf2 has been considered as the most important signaling pathway in the regulation of the oxidative stress of animals [19,20]. In addition, AFB1 can impair the function of liver mitochondria by activating the second messengers in this pathway, such as B-cell Leukemia/Lymphoma-2 associated X protein (Bax ) and Ca^2+^, which can release cytochrome C (Cyt-C), apoptotic protease activating factor-1 (Apaf-1) and caspase9 complexes, and then activate caspase3, 6 and 7, causing apoptosis of the liver [21].

Res is a non-flavonoid polyphenol compound widely prevalent in various plants, including grape, peanut and roe, or its fruit [22,23]. It has many biological functions such as antioxidant, anti-inflammatory, antibacterial and antiviral properties, and it contributes to the regulation of cell metabolism [24,25]. Res has previously shown a significant effect regarding oxidative stress in the liver by, for example, decreasing levels of liver enzymes (ALT, AST and ALP) in broiler chickens, increasing the activity of antioxidants, including glutathione S—transferase, glutathion reductases, glutathione peroxidase, superoxide dismutase, catalase, (GST, GR, GPx, SOD and CAT) [26,27], removing N6-methyl adenosine (M6A) from mice treated with ROS, and accelerating the metabolism of AFB1 [28]. Res was shown to significantly increase the expression of NAD (P) H quinone oxidoreductase 1 (NQO1), beta-glutamyl cysteine synthase and heme oxygenase-1 (HO-1) genes, in the endothelial cells of coronary arteries, thereby reducing inflammatory stimulation and oxidative stress [29]. However, there are few reports regarding the effect of dietary Res on liver damage induced by AFB1 and its underlying mechanism is not clear [26,28]. Therefore, the purpose of this study was to assess the role of Res in the potential prevention of acute liver injury induced by AFB1 and to further investigate the role of the Nrf2 and apoptotic pathways in liver protection in ducks.

## 2. Materials and Methods

### 2.1. Animals and Experimental Design

This study was approved by the Science Ethics Committee of Northeast Agricultural University (Moral approval code: NEAU-[2011]-9). Res (purity ≥ 98%) was purchased from Nanjing NiuCuiBao Biological Technology (Nanjing, China). AFB1 (purity ≥ 98%) was purchased from Shanghai Yuanye Bio-Technology Co, Ltd. (Shanghai, China). A total of 270 one-day-old, male, specific pathogen free (SPF) ducks (33.8 ± 0.2 g) from the same incubator was randomly divided into three groups with 6 repetitions and 15 ducks per repetition. The three groups, the Control group, the AFB1 group and the AFB1 + Res group, were fed a basic diet, a basic diet, and a basic diet containing 500 mg/kg Res, respectively. The basal diets were formulated in accordance with the National Research Council (1994), and the ingredient composition was identical to that shown in Table 1. On the 70th day, the ducks in the AFB1 group and the AFB1 + Res group were given 60 μg of AFB1/kg of body weight by gavage, followed by fasting for 12 h.

### 2.2. Sample Collection

After being fasted for 12 h, 6 ducks per replicate were randomly selected in order to collect blood from their wing veins. Ten-milliliter blood samples collected in heparin tubes were centrifuged at 1000× *g* for 15 min at 4 °C. The plasma was separated and stored at −20 °C before it was used. Six ducks per replicate were killed after being anesthetized by the inhalation of ether, and samples from the left lateral lobe of the liver of each duck were collected. Samples for use in qRT-PCR analysis, protein extraction, and antioxidant capacity analysis, were immediately placed in liquid nitrogen and stored at −80 °C, and samples were fixed in 4% neutral buffered formalin or 2.5% glutaraldehyde- phosphate-buffered saline (PBS) for histopathological analysis or ultrathin sectioning, respectively.

### 2.3. Histopathological Analysis

Rapid collection was carried out for 0.125 cm^3^ samples of duck liver, which were then fixed with a 4 % paraformaldehyde solution for 72 h and processed using routine histological techniques. After paraffin embedding, 5 μM sections were cut and stained with hematoxylin and eosin (HE) to assess the tissue structure. The slides were visualized using a light microscope (Nikon Eclipse Ci-L, Tokyo, Japan). The liver tissue was immobilized using 2.5% glutaraldehyde-PBS for 1 week and then sequentially treated with 1% osmic acid, dehydrated, and embedded in resin. Finally, ultrathin sections were stained with 100 uranyl acetate and lead citrate before examination using TEM (H-7650, Hitachi, Tokyo, Japan).

### 2.4. Measurement of AFB1-DNA Adduct in Liver Genomic DNA

Liver homogenate was prepared in Stroke-physiological saline solution (4 °C, 0.9% NaCl, pH = 7.2) and centrifuged at 5000× *g* for 10 min at 4 °C to obtain the supernatant. The content of AFB1-DNA adduct in the supernatant was determined by means of a competitive ELISA kit (Jiangsu Jingmei Biotechnology Ltd., Yancheng, China) used in accordance with the manufacturer’s instructions. The optical density (OD) values were read at a wavelength of 450 nm, and the content of AFB1-DNA adduct was calculated according to the standard curve.

### 2.5. Measurement of Total P450 Content in Liver

The content of CYP450 in the liver supernatant was determined using duck cytochrome P450 (CYP450) ELISA kit (No. H303) (Nanjing Jiancheng Bioengineering Institute, Nanjing, China), and the manufacturer’s instructions were strictly followed. The OD value was determined at a wavelength of 450 nm and the content of the CYP450 complex was calculated according to the standard curve.

### 2.6. Measurement of Antioxidant Indices of Liver

Liver tissue, homogenized to a 10% liver homogenate solution in precooled 0.85% normal saline, was used in tests to determine activity tests. After the protein concentration of the liver suspension was determined using a Coomassie brilliant blue kit, GST, reduced glutathione (GSH), SOD, CAT, H_2_O_2_ and malondialdehyde(MDA)contents were measured using commercial kits from Nanjing Jiancheng Bioengineering Institute (Nanjing Jiancheng Bioengineering Institute, Nanjing, China), following the manufacturer’s instructions.

### 2.7. Plasma Biochemical Analysis

The plasma biochemical factors related to hepatic damage, including alanine aminotransferase (ALT), aspartate aminotransferase (AST), alkaline phosphatase (ALP), total bilirubin (TBIL), albumin (ALB), globulin (GLO) and lactate dehydrogenase (LDH), were measured with an automatic biochemical analyzer (Roche Cobus Mira Plus, Roche, Basel, Switzerland) using commercial diagnostic kits (Nanjing Jiancheng Bioengineering Institute, Nanjing, China)

### 2.8. RNA Isolation and Quantitative Real-Time PCR Analysis (qPCR)

Total RNA was extracted from the liver samples with an RNAiso Plus kit (Takara, Japan). The quantity and quality of the total RNA were determined with a spectrophotometer (Implen Nanophotometer P-330, Implen, Munich, Germany) to ensure that the A260/A280 ratio was 1.8–2.0. First-strand cDNA was synthesized from 1 μg of isolated RNA using a PrimeScript™ RT reagent kit with a gDNA Eraser (Takara Bio Inc., Shiga, Japan), following the manufacturer’s instructions. The expression patterns of the targeted genes were determined via qRT-PCR using the SYBRVR Premix Ex TaqTM Kit (Takara Biotechnology Co, Ltd., Dalian, China) in ABI Quant Studio 3 (Applied Biosystems, Waltham, MA, USA). In this study, the primers used were procured from Sangon Biotech Co, Ltd. (Shanghai, China), as shown in Table 2. An amount of 20 μL of PCR reaction solution was composed of 0.5 μL of forward primer (final concentration: 0.5 μmol/L) and 0.5 μL of reverse primer (final concentration: 0.5 μmol/L), 10 μL of qPCR master mix, 1 μL of cDNA template and 8 μL of H_2_O. The PCR conditions were: 94 °C for 5 min; 30 cycles of 94 °C for 30 s, 55 °C for 30 s and 72 °C for 60 s, followed by a final elongation step at 72 °C for 10 min. The housekeeping gene β-actin was used as a reference gene for normalization. Data were analyzed according to the 2^−∆∆Ct^ method, the relative mRNA levels were calculated according to the mean ^−∆∆^Ct values from each group.

### 2.9. Preparation of Protein Extracts and Western Blot Analysis 

The total protein from the liver tissue was extracted with a superactive RIPA lysis buffer (Beyotime Biotechnology, Shanghai, China). The protein concentration of the extracted protein was determined using a BCA analysis kit (Beyotime Biotechnology, Shanghai, China). Protein extract was subjected to 12% SDS-PAGE (30 μg per lane) and transferred to polyvinylidene difluoride membrane in standard tris-glycine transfer buffer (pH 8.3, 0.5% SDS). Then, the membranes were blocked for 1 h at room temperature in PBST containing 5% non-fat milk powder and incubated overnight at 4 °C with primary antibodies, including GAPDH mouse monoclonal antibody (1:750 dilution), Sirt1 rabbit monoclonal antibody (RmAb) (1:1000 dilution), NF-κB-p65 rabbit polyclonal antibody (RpAb) (1:1000 dilution), Nrf2 RpAb (1:1000 dilution), Keap1 RpAb (1:1000 dilution) (Beyotime Biotechnology, Shanghai, China, and the catalog numbers were AF5009, AF1267, AF0246, AF7623 and AF7335, respectively), HO-1 RpAb (1:500 dilution), Bcl-2 RpAb (1:500 dilution), Bax RpAb (1:500 dilution), cleaved caspase-3 RpAb (1:500 dilution), cleaved caspase-9 RpAb (1:500 dilution) and Cyt-c RpAb (1:500 dilution) (Wanleibio, Shenyang, China, and the catalog numbers were WL02400, WL01556, WL01637, WL02117, WL01838 and WL02410, respectively) antibodies. After being washed with TBST 3 times, the membranes were incubated in the corresponding secondary antibodies (HRP-labeled goat anti-mouse IgG or HRP-labeled goat anti-rabbit IgG, 1:1000 dilution) (Beyotime Biotechnology, Shanghai, China, A0216 or A0208) for 2 h at 37 °C, and then were washed with TBST 3 times. Western blot images were quantified by measuring the intensity of correctly sized bands using Alpha Imager 2200 (Alpha Innotech Corporation, San Jose, CA, USA), and all protein measurements were normalized to GAPDH. The original western blot figures are showed in supplementary materials (Figure S1).

### 2.10. Statistical Analysis 

The data were analyzed via one-way ANOVA using SPSS 22.0 (IBM-SPSS Inc, Chicago, IL, USA). The LSD test was used as a post hoc test, and data are expressed as the means and standard errors (SEMs) of means. When p < 0.05, the difference was considered significant.

## 3. Results

### 3.1. Effect of Res on the Liver Microstructure and Ultrastructure of Duck Exposed in AFB1 

**Figure 1:**
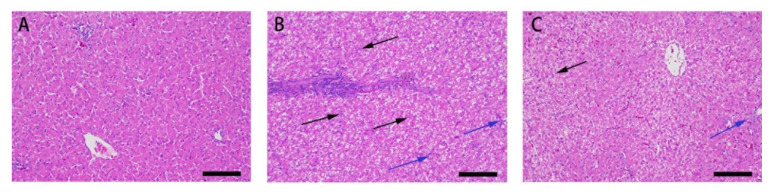
Effect of Res on the microstructure of duck liver exposed to AFB1 (magnification 200×). (**A**) the control group; (**B**) the AFB1 group; (**C**) the AFB1 + Res group. The black arrowheads indicate swollen liver cells, and the blue arrowheads indicate inflammatory cell infiltration.

The results of HE staining of ducks’ livers are shown in Figure 1.

In the control group, the hepatic lobules were intact and the cell morphology was normal. The structure of the hepatic lobules was complete, the hepatocytes were arranged in an orderly manner, and the nucleus was clearly visible (Figure 1A). In AFB1 group, the hepatocytes were disordered with respect to the amount of inflammatory cell infiltration and displayed slight steatosis, and vacuolization of hepatocytes and residual hepatocytes in necrotic areas was observed (Figure 1B). Compared with the AFB1 group, hepatocytes in the Res + AFB1 group were arranged in an orderly manner, vacuolization was obviously reduced and the amount of necrotic cells were decreased (Figure 1C).

**Figure 2:**
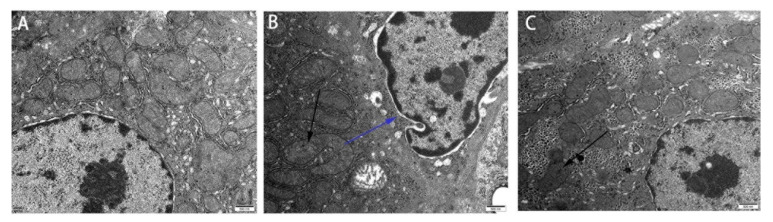
Effect of Res on the ultrastructure of liver of duck liver exposed to AFB1 (500 nm). (**A**) the control group; (**B**) the AFB1 group; (**C**) the AFB1 + Res group. The blue arrowheads indicate the damage to hepatocyte nuclear membrane, the black arrowheads indicate mitochondria swollen irregularly and their cristae fractured and fuzzy.

The effect of Res on the ultrastructure of ducks’ livers exposed to AFB1 was examined by using SEM, and the results are shown in Figure 2. The ultrastructure of liver in the control group was normal, which showed that the hepatocytes were arranged closely, and the structure and morphology of the nuclei, mitochondria and chromatin were all normal (Figure 2A). The liver in the AFB1 group showed the pathological characteristics of microstructure, including damage to the hepatocyte nuclear membrane and mitochondrial membrane, vacuolization of nuclei and mitochondria, swelling of the mitochondria, and reduction in cristae number (Figure 2B). Res supplementation alleviated the ultrastructural alteration caused by AFB1. In the Res + AFB1 group, the changes with respect to the hepatocyte morphology, nuclei and mitochondrial cristae were reduced compared to those of the AFB1 group (Figure 2C).

### 3.2. Effect of Res on Liver Function Impaired by AFB1

The effect of Res supplementation in the diets of ducks on liver function impaired by AFB1 was as shown in Table 3. Compared with the control group, the concentration of aminotransferase (ALT) was significantly increased (*p* < 0.05), and the concentrations of total protein (TP) and globulin (GLO) were significantly decreased (*p* < 0.05) in both the AFB1 and AFB1 + Res group. The concentration of lactate dehydrogenase (LDH) in the AFB1 group was significantly increased (*p* < 0.05) and the ALB concentration in the AFB1 + Res group was significantly decreased (*p* < 0.05) compared with the control group. There was no significant change (*p* > 0.05) in the concentrations of aspartate aminotransferase (AST), alkaline phosphatase (ALP), and total bilirubin (TBIL) in plasma, among the three groups. Compared with the AFB1 group, the contents of ALT, AST, ALP, TBIL, ALB, GLO and LDH in the Res + AFB1 group were decreased, but did not reach statistical significance (*p* > 0.05).

### 3.3. Effect of Res on the Liver Antioxidation Status of Ducks Exposed to AFB1 

As shown in Table 4, compared with the control group, AFB1 significantly decreased the activity of total antioxidant capacity (T-AOC), CAT and SOD in ducks’ livers (*p* < 0.05), whereas it increased the content of H_2_O_2_ (*p* < 0.05). Compared with the AFB1 group, the contents of SOD, T-AOC and CAT were significantly increased (*p* < 0.05), and the content of H_2_O_2_ was decreased in the AFB1 + Res group (*p* > 0.05).

### 3.4. Effect of Res on the Content of AFB1-DNA Adduct and CYP450 Content in the Ducks’ Livers and Plasma Exposed to AFB1

**Figure 3:**
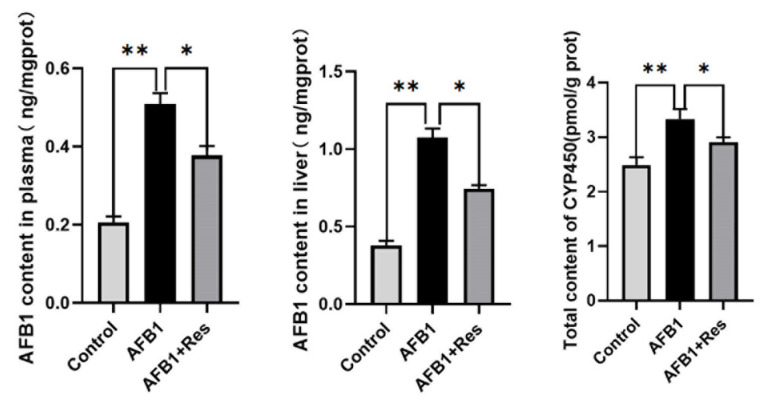
Effect of Res on the content of AFB1-DNA adduct and CYP450 content in the duck liver and plasma exposed to AFB1. Effect of Res on the content of AFB1-DNA adduct and CYP450 content in the duck liver and plasma exposed to AFB1. Values are expressed as Mean ± SEM (*n* = 6), and * means *p* < 0.05, ** means *p* < 0.01.

In hepatocytes, AFB1 can be transformed into AFB1–8,9-epoxide by phase- I metabolic enzyme cytochromes P450 (CYP450), which can form AFB1–8,9-epoxide-DNA adducts with DNA. Therefore, the content of the intermediate toxic metabolite of AFB1 (AFB1-DNA adduct) and the mRNA levels of the CYP450 genes were determined. In duck plasma and liver, the content of AFB1-DNA adduct in the AFB1 group was very significantly higher than that of the control group (*p* < 0.01), and Res supplementation significantly decreased the level of AFB1-DNA adducts compared with that of the AFB1 group (*p* < 0.05) (Figure 3). As shown in Figure 3, AFB1 challenge significantly increased the total CYP450 content (*p* < 0.01). Res supplementation in the diet of ducks significantly decreased the CYP450 content (*p* < 0.05).

### 3.5. Effect of Res on the Expression of Phase-I Metabolic Enzyme CYP450 in AFB1 

**Figure 4:**
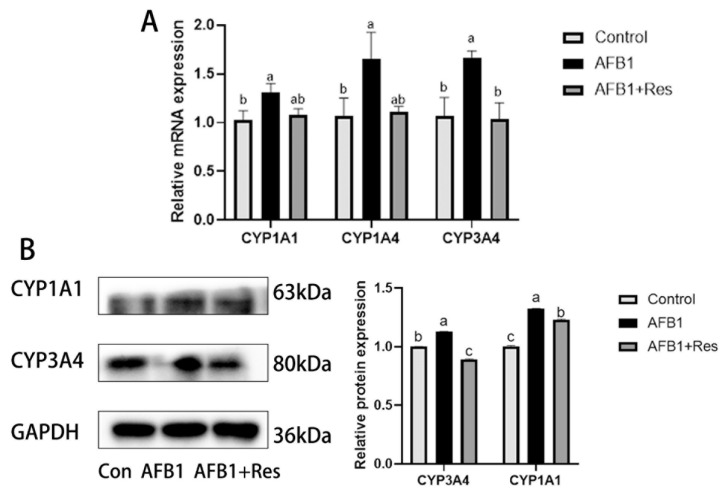
Expression of phase I metabolizing enzyme in the duck liver exposed to AFB1. (**A**): mRNA levels of the related genes of phase- I metabolic enzymes. (**B**): protein levels of the related genes of phase- I metabolic enzymes. Values are represented as the mean ± SEM (*n* = 6). ^a–c^ Mean values with same superscript letters or no letters within a row were of no significant difference (*p* > 0.05), those with different superscript letters were of significant or extremely significant difference (*p* < 0.05).

As shown in Figure 4, compared with the control group, the mRNA relative expression levels of CYP1A1, CYP1A4 and CYP3A4 genes (Figure 4A) and protein expression levels of CYP1A1 and CYP3A4 (Figure 4B) in the liver were significantly increased (*p* < 0.05) in the AFB1 group. The supplementation of Res in the ducks’ diets significantly decreased the mRNA relative expression of the CYP3A4 gene and protein expression levels of CYP1A1 and CYP3A4 (*p* < 0.05) compared with the AFB1 group.

### 3.6. Effect of Res on GSH Content and mRNA Expression of Related Regulatory Genes in Liver of AFB1-Exposed Duck 

**Figure 5:**
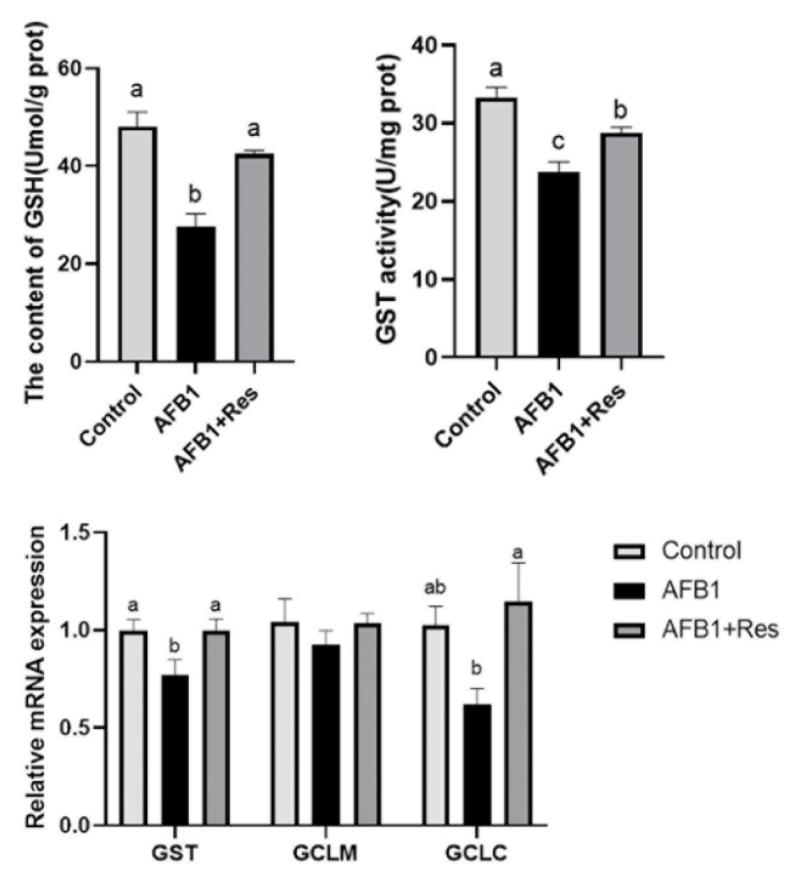
Effect of Res on the GSH content and expression of its regulated genes in the duck liver exposed to AFB1. Values are represented as the mean ± SEM (*n* = 6). ^a–c^ Mean values with same superscript letters or no letters within a row were of no significant difference (*p* > 0.05), those with different superscript letters were of significant or extremely significant difference (*p* < 0.05).

GSH is a cofactor that mediates the detoxification of GST and is conducive to the metabolism of toxic substances in the liver. GSH synthesis is regulated by GCLC and GCLM in the liver. As shown in Figure 5, there was no significant difference in the mRNA levels of the GCLM gene in livers among the control group, the AFB1 group and the AFB1 + Res group. Compared with the control group, AFB1 exposure significantly decreased GSH content (*p* < 0.05), GST activity (*p* < 0.01), and mRNA levels of genes (GST) (*p* < 0.05) in the liver in the AFB1 group. Compared with the AFB1 group, the GSH content, GST activity and the mRNA levels of GST and GCLC genes were significantly increased in the AFB1 + Res group (*p* < 0.05).

### 3.7. Effect of Res and AFB1 on Nrf2 Signaling Pathway in Liver

**Figure 6:**
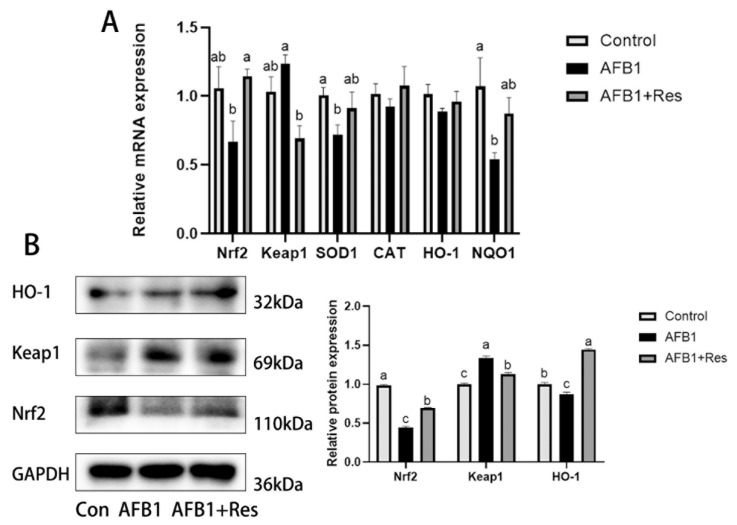
Effect of Res on Nrf2 signaling pathway in duck liver exposed to AFB1. (**A**): mRNA levels of the related genes of Nrf2 signaling pathway. (**B**): protein levels of the related genes of Nrf2 signaling pathway. Values are represented as the mean ± SEM (*n* = 6). ^a–c^ Mean values with same superscript letters or no letters within a row were of no significant difference (*p* > 0.05), those with different superscript letters were of significant or extremely significant difference (*p* < 0.05).

As shown in Figure 6A, dietary Res and AFB1 exposure had an obvious influence on the Nrf2 signaling pathway in ducks’ livers. Compared with the control group, the mRNA levels of the Nrf2 genes and the downstream genes of SOD1and NQO1 were significantly decreased in the livers of the AFB1 group (*p* < 0.05), while CAT and HO-1 gene mRNA levels displayed a downward trend (*p* > 0.05). Compared with the AFB1 group, the mRNA levels of the Nrf2 and NQO1 genes were significantly increased in the AFB1 + Res group (*p* < 0.05), while that of Keap1, SOD1, CAT and HO-1 displayed an upward trend (*p* > 0.05).

As shown in Figure 6B, the protein concentration of Nrf2, Keap1 and HO-1 in the liver was determined via Western Blot. AFB1 exposure significantly reduced the protein levels of Nrf2 (*p* < 0.01) and HO-1 (*p* < 0.05), and significantly increased Keap1 protein (*p* < 0.05). Meanwhile, dietary Res significantly improved protein levels of Nrf2 (*p* < 0.05) and HO-1 (*p* < 0.01) and significantly inhibited Keap1 protein levels (*p* < 0.01) in ducks’ livers exposed to AFB1.

### 3.8. Effect of Res on Inflammatory Response of Duck Induced by AFB1

**Figure 7:**
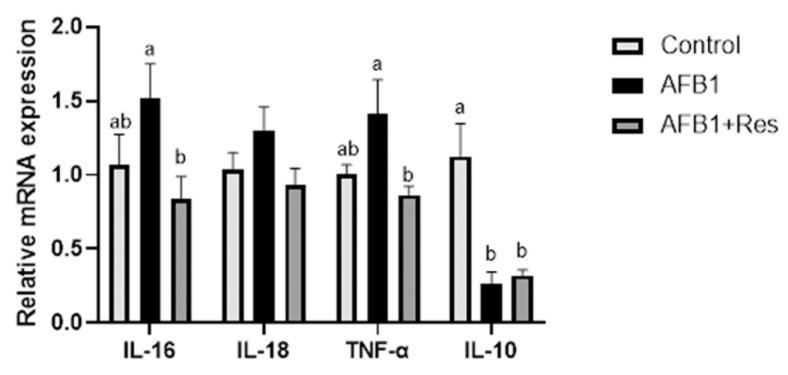
Effect of Res on the expression of inflammatory factor genes in duck liver exposed to AFB1. Values are represented as the mean ± SEM (*n* = 6). ^a,b^ Mean values with same superscript letters or no letters within a row were of no significant difference (*p* > 0.05), those with different superscript letters were of significant or extreme significant difference (*p* < 0.05).

In addition, the mRNA of several inflammatory cytokine genes in liver was investigated using qRT-PCR, and the results are shown in Figure 7. Compared with the control group, the mRNA levels of pro-inflammatory cytokines IL-16, IL-18 and TNF-α in the AFB1 group were down-regulated. IL-18 displayed an upward trend, but the difference was not significant (*p* > 0.05), and the mRNA levels of anti-inflammatory cytokines IL-10 were significantly decreased *(p* < 0.05). Compared with the AFB1 group, the addition of dietary Res down-regulated the mRNA levels of IL-16 (*p* < 0.05), TNF-α (*p* < 0.05) and IL-18 (*p* > 0.05), while the level of IL-10 mRNA level was down-regulated (*p* > 0.05).

### 3.9. Effect of AFB1 and Res on the Expression of Sirt1 and NF-κB in Liver

**Figure 8:**
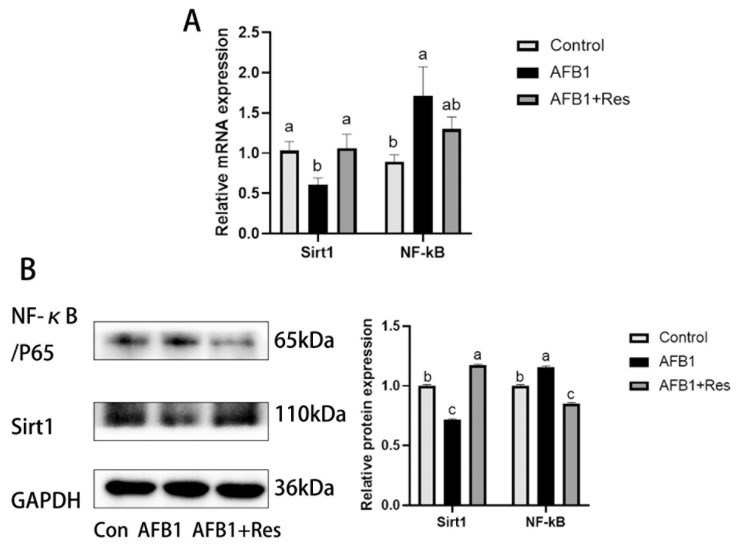
Effect of Res on the expression levels of Sirt1 and NF-κB in duck liver exposed to AFB1. Values are represented as the mean ± SEM (*n* = 6). ^a–c^ Mean values with same superscript letters or no letters within a row were of no significant difference (*p* > 0.05), those with different superscript letters were of significant or extremely significant difference (*p* < 0.05). (**A**) mRNA levels of Sirt1 and NF-κB genes. (**B**) protein levels of Sirt1 and NF-κB genes.

As shown in Figure 8, compared with the control group, AFB1 exposure significantly decreased Sirt1 expression and significantly increased NF-κB expression both in mRNA and protein levels (*p* < 0.05). The mRNA and protein levels of Sirt1 in the AFB1 + Res group were both significantly higher than those in the AFB1 group (*p* < 0.05). Dietary Res very significantly reduced the protein level of NF-κB (*p* < 0.01) significantly increased by AFB1, although it had no significant effect on the mRNA level of the NF-κB gene.

### 3.10. Effect of Res on Mitochondrial Apoptosis Pathway Induced by AFB1 

**Figure 9:**
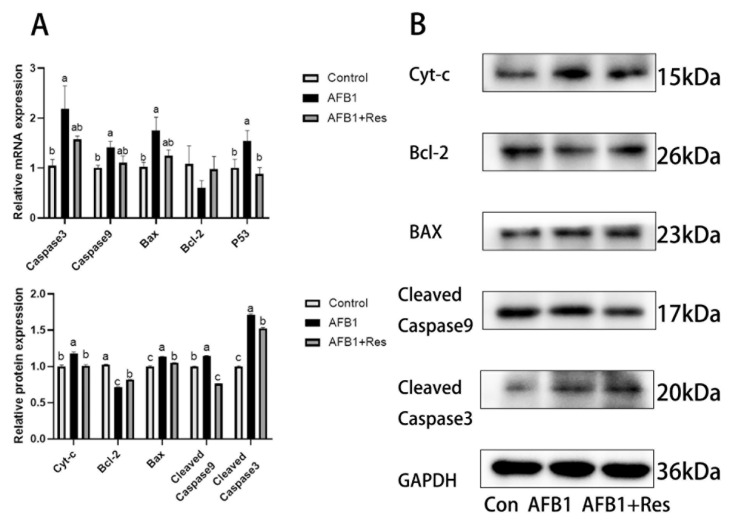
Effect of Res on the expression level of apoptosis-related genes in duck liver exposed to AFB1. Values are represented as the mean ± SEM (*n* = 6). ^a–c^ Mean values with same superscript letters or no letters within a row were of no significant difference (*p* > 0.05), those with different superscript letters were of significant or extremely significant difference (*p* < 0.05). (**A**) mRNA levels of apoptosis-related genes. (**B**) protein levels of apoptosis-related genes.

To investigate the effect of Res on the mitochondrial apoptosis pathway induced by AFB1, the expression levels of a series of genes related to the pathway were determined using qRT-PCR and Western Blot. As shown in Figure 9A, in the AFB1 group, the mRNA levels of pro-apoptotic Caspase3, Caspase9, P53 and Bax were all significantly higher than those in the control group (*p* < 0.05), except that for the mRNA level of Bcl-2, which was decreased (*p* > 0.05). Compared with AFB1 treatment, the mRNA level of P53 in the AFB1 + Res group was significantly decreased (*p* < 0.05), and the mRNA levels of Caspase3, Caspase9 and Bax showed a downward trend (*p* > 0.05), while the mRNA level of Bcl-2 showed a downward trend (*p* > 0.05). As expected, the dietary supplementation of Res significantly reduced the protein levels of cleaved caspase-3 (*p* < 0.05), cleaved caspase-9 (*p* < 0.01), Cyt-c (*p* < 0.05) and Bax (*p* < 0.05) increased by AFB1, while it significantly improved the protein level of Bcl-2 (*p* < 0.05) inhibited by AFB1 (Figure 9B).

## 4. Discussion

The main finding of this study was that Res played an important precautionary and protective role against AFB1-induced liver damage in ducks. In our previous study, dietary supplementation with 400 mg/kg Res did not improve growth performance [30]. In this study, a similar effect of dietary Res (500 mg/kg) on growth performance was found, and Res did not significantly influence growth performance, including daily rates, feed intake and feed efficiency (data not shown). In this study, the exposed AFB1 duck liver edema, structural damage, and vacuolization of hepatocytes, nuclei, mitochondria, and other organelles showed different degrees of abnormalities. Nevertheless, the above changes after Res treatment were significantly reduced and tended to be normal. This suggests that Res ameliorates acute liver injury induced by AFB1. AFB1 can induce oxidative stress to alter the activity of antioxidant enzymes, deplete cellular antioxidant systems, such as SOD, GPx and CAT, and enhance free radical production [31,32]. The supplementation of 1% Res significantly increased the contents of T-AOC, CAT and SOD in the plasma of broilers [26]. Studies have shown that Res can protect mice from AFB2 injury, mainly in relation to the scavenging activity of superoxide, H_2_O_2_ and 1,1-diphenyl-2-picrylhydrazyl (DPPH) free radicals, and the antioxidant effect [33]. Our findings demonstrated that AFB1 led to significant oxidative damage and Res reversed the trend, as shown with the decrease in GSH, GSH-ST, T-AOC, CAT and SOD levels decrease and the increase in H_2_O_2_ and MDA levels. Therefore, Res may have a protective effect on AFB1-induced oxidative damage.

AFB1 is a precursor carcinogen, and its toxicity is mediated by the CYP450 enzyme system into AFBO [12]. AFBO can directly immobilize large cell molecules, including nucleic acids and proteins, leading to excessive ROS production and reduced GST activity and GSH content. CYP450 enzymes are involved in the metabolism of AFB1 in a variety of poultry [34]. There are more than 50 CYP450 enzymes, and these are predominantly expressed in the liver, but several enzymes of this class, including CYP1A2, CYP2C9, CYP3A4, and so on, metabolize 90 percent of drugs [35]. It has been found that the content of CYP 450 increased in the livers of the AFB1 group, and the levels of CYP1A1, CYP1A2, CYP2A6 and CYP3A4 mRNA increased significantly [13,36]. Because these enzymes are responsible for the biological activation of AFBO, inhibiting these enzyme activities may reduce the production of AFBO. Our study showed that Res reduced the formation of AFB1-DNA adducts by inhibiting the activity of reductase and regulating the function of three CYP450 enzymes (CYP1A1, CYP1A4 and CYP3A4), which demonstrated that Res resisted the hepatotoxicity of AFB1 by inhibiting the biotransformation induced by the I-phase enzyme.

AFB1 is a cytotoxic substance that leads to toxic metabolites and excessive ROS, inhibits the function of the antioxidant system, and thus induces oxidative stress in liver cells [36]. Nrf2 is a nuclear transcription factor that regulates the body’s phase-II detoxification enzyme system and antioxidant system, and plays an important role in the metabolism of exogenous toxic substances and resistance to oxidative stress [37].

When oxidative stress occurs, reactive oxygen activates the antioxidation pathway of Nrf2, whose phosphorylation leads to its dissociation from Keap1 and subsequent translocation to the cell nucleus, where it acts with the anti-oxidant reaction element (ARE), regulates the transcription of the ARE and antioxidant enzymes downstream of the gene, and provides sufficient levels of antioxidants to reduce the formation of ROS and protect the body from liver cell damage [38]. The main route of AFB1 detoxification is binding with phase-II metabolic enzymes such as glutathione (GST), glucuronate and sulfonate. GSH is the first line of defense against ROS and can reduce the toxicity of AFB1 by forming an AFBO-GSH conjugate. It was shown that the continuous feeding of broilers with a diet that included 5 mg/kg of AFB1 for 28 days significantly inhibited the activity and mRNA level of the liver GST gene [39]. AFB1 was shown to inhibit the Nrf2 pathway and further reduce phase-II detoxification, such as HO-1, NQO1, while mice renally treated with Res displayed reduced production levels of reactive oxygen species and raised HO-1 levels [40]. Res protected primary rat hepatocytes from oxidative stress by increasing Nrf2 levels and inducing their translocation to the nucleus [41]. In this study, the results showed that Res alleviated the inhibition of the Nrf2 pathway in ducks’ livers caused by AFB1 exposure, increased the mRNA expression of related genes, enhanced GST-mediated phase-II metabolism and antioxidant capacity, inhibited the accumulation of toxic products (AFBO and ROS) in the liver and, finally, protected the liver from the toxic effects of AFB1.

Sirtuin1 (Sirt1) regulates the acetylation of various proteins, such as NF-κB, p53 and FoxOs, through its deacylase activity, thus regulating the life span, inflammation, senescence, and metabolism of different organisms [42,43]. Res induces the increased expression of Sirt1, which inhibits the activity of the pro-apoptotic protein Bax and promotes the expression and activity of the anti-apoptotic protein Bcl-2 by regulating the deacetylation of the P53 lysine site, thereby inhibiting the apoptosis of cardiomyocytes [44]. Sirt1 interacts with the RelA/p65 subunit of NF-κB and deacetylates its Lys310 residue, which is essential for NF-κB transcription activity [45]. As a Sirt1 agonist, Res significantly increases Sirt1 activity through a heterogeneous interaction [46]. Res activates Sirt1 and then causes NF-κB down-regulation, which can eliminate colitis. The main cellular mechanism of Res resistance to dextran sodium-sulphate-induced colitis is achieved by down-regulating the expression of inflammatory molecules such as IL-6, IL-1β, IFN-α and TNF-α [47]. Knocking out the Sirt1 gene activated NF-κB and increased the secretion of pro-inflammatory cytokines, while Res inhibited the production of inflammatory factors mediated by NF-κB in vitro and in vivo [48]. In addition, Res has been shown to modulate the deacetylation of NF-κB via Sirt1 activation, as well as the TNF-α induced inflammation of human chondrocytes [49]. In this study, the mRNA and protein levels of Sirt1 activity were inhibited, and the NF-κB expression level was increased in AFB1 hepatic tissues. Our results suggest that Res may ameliorate AFB1-induced liver damage by up-regulating the expression of Sirt1 in liver cells.

Apoptosis is programmed cell death, which is controlled by genes that regulate cell deaths by removing diseased or damaged cells [50]. AFB1 impairs mitochondrial function, leads to membrane structure damage and increased permeability, reduces the ability to regulate ion balance across the membrane, and ultimately causes membrane potential decline. The release of Cyt-C into the cytoplasm is a hallmark of mitochondria-mediated apoptosis [51]. Apoptosis is mainly regulated by intracellular apoptotic proteins, including anti-apoptotic Bcl-2 and Bcl-xl, and pro-oxidant Bad and Bax. Pro-caspase9 is the promoter of the mitochondrial apoptosis pathway. After Cyt-C is released by mitochondria, pro-Caspase9 can bind to Cyt-C and the signaling connector molecule Apaf-1 and form a complex. At the same time, pro-caspase9 itself is cleaved to caspase9. Cleaved caspase9 further activates downstream apoptotic executioner caspase3 to perform a series of cascade reactions, resulting in the occurrence of apoptosis [52,53]. P53 is an apoptotic protein. Activated P53 can transfer to mitochondria and act directly on Bcl-2, inhibit the binding ability of Bcl-2 to Bax, and promote the influx of Cyt-C from the nucleus to the cytoplasm [54]. In the present study, AFB1 exposure induced hepatocyte apoptosis by increasing the expression of Bax, Caspase3 and P53 genes, and by decreasing Bcl-2 expression. The mitochondria are the main attack targets of the ROS produced in the process of AFB1 metabolism in hepatocytes. Apoptosis mediated by mitochondrial damage is suggested to be related to AFB1-induced oxidative stress induced by AFB1. In our study, Res supplementation inhibited the expression levels of pro-apoptotic genes (e.g., Bax, P53, Caspase3, Caspase9, Cyt-C) and improved the expression levels of the anti-apoptotic gene Bcl-2 in hepatocytes, which demonstrated that dietary Res implemented the effective inhibition of hepatocyte apoptosis caused by AFB1.

## 5. Conclusions

**Figure 10:**
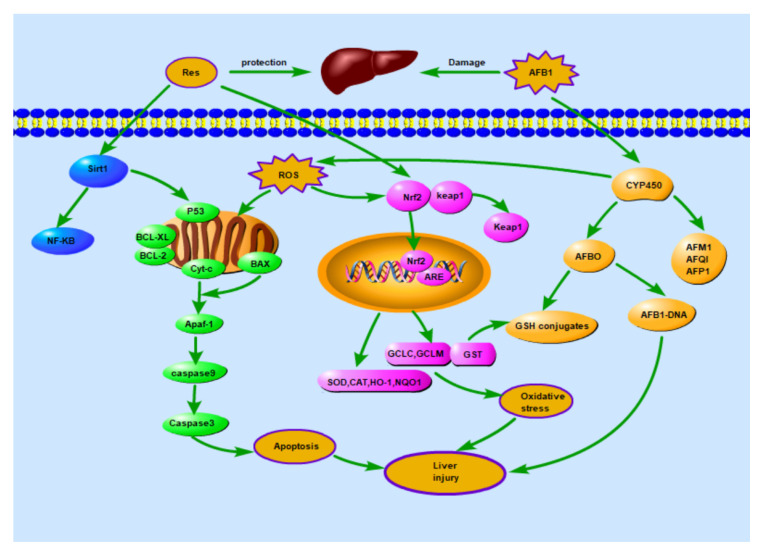
Res activated the Nrf2 pathway and inhibited mitochondrial apoptosis pathway to alleviate liver damage induced by AFB1.

This study showed that the supplementation of 500 mg/kg of Res in ducks’ diets inhibited the level of acute liver injury of ducks exposed to AFB1, and dietary Res effectively alleviated the apoptotic pathway by inhibiting the metabolism of AFB1, up-regulating the activity of phase-II metabolism enzymes, activating the expression of Nrf2 and Sirt1, down-regulating the activities of NF-κB, and suppressing the cell apoptosis pathway (Figure 10). Therefore, Res may serve as a potential phytochemical for use in the treatment of acute AFB1 toxicity in ducks and other animals.

## Figures and Tables

**Table 1:** Ingredient composition and nutrient content of the basal diet (%, as-fed basis).

Item	1–4 Weeks	5–8 Weeks	9–10 Weeks
Ingredient			
Corn (7.9)	61.70	68.94	75.80
Soybean meal (45)	26.09	26.80	20.10
Corn protein flour (55)	7.90	—	—
Dicalcium phosphate	1.40	1.40	1.40
Limestone	1.08	1.06	1.06
Salt	0.38	0.38	0.38
DL-Methionine	0.15	0.22	0.16
L-Lysine	0.20	0.10	0.00
choline chloride (50%)	0.10	0.10	0.10
Premix	1.00 ^1^	1.00 ^2^	1.00 ^3^
Total	100	100	100
Nutritional level			
Calculated nutrient ^4^			
Metabolizable energy (MJ/kg)	12.14	11.98	12.21
CP (%)	20.67	17.51	15.03
Calcium (%)	0.9	0.9	0.88
Total phosphorus (%)	0.68	0.67	0.65
Non-phytate phosphorus (%)	0.44	0.44	0.44
Lysine (%)	1.07	0.95	0.71
Methionine (%)	0.48	0.48	0.39
Methionine +cystine (%)	0.81	0.75	0.63
Threonine (%)	0.75	0.66	0.56
Tryptophane (%)	0.21	0.19	0.16

^1^ The premix provided per kilogram diet: vitamin A 4000 IU, vitamin D3 2000 IU, vitamin E 20 mg, vitamin K3 2.0 mg, vitamin B1 2.0 mg, vitamin B2 12 mg, vitamin B6 3.0 mg, vitamin B12 0.02 mg, nicotinic acid 50 mg, D-pantothenic acid 10 mg, folic acid 1 mg, biotin 0.2 mg, Cu 8 mg, Fe 60 mg, Mn 100 mg, Zn 60 mg, Se 0.2 mg, I 0.4 mg. ^2^ The premix provided per kilogram diet: vitamin A 3000 IU, vitamin D3 2000 IU, vitamin E 10 mg, vitamin K3 2.0 mg, vitamin B1 1.5 mg, vitamin B2 8 mg, nicotinic acid 30 mg, D-pantothenic acid 10 mg, vitamin B6 3.0 mg, vitamin B12 0.02 mg, biotin 0.1 mg, folic acid 1 mg, Cu 8 mg, Fe 60 mg, Mn 80 mg, Zn 40 mg, Se 0.2 mg, I 0.4 mg. ^3^ The premix provided per kilogram diet: vitamin A 2500 IU, vitamin D3 1000 IU, vitamin E 10 mg, vitamin K3 2.0 mg, vitamin B1 1.5 mg, vitamin B2 8 mg, nicotinic acid 30 mg, D-pantothenic acid 10 mg, vitamin B6 3.0 mg, vitamin B12 0.02 mg, biotin 0.1 mg, folic acid 1 mg, Cu 8 mg, Fe 60 mg, Mn 80 mg, Zn 40 mg, Se 0.2 mg, I 0.3 mg. ^4^ Values were calculated based on the data provided by Feed Database in China (2004).

**Table 2:** Primer sequences and product lengths of target gene fragments.

	Transcripts	Sequence (5′–3′)	Product Size (bp)
Oxidative	Nrf2	F: GGCCGCCTTGAAGCTCATCTC	177
stress		R: CTTGCCTCTCCTGCGTATATCTCG	
	Keap1	F: TTTTCAAGACCTCACCCTCCATAAACC	113
		R: AGTAGCCCAAGGACTGCCGATAG	
	HO-1	F: AGAGCCAGGAGAACGGTCACC	114
		R: TGCTTGTCCTCTCGCTTTCTTTCC	
	CAT	F: ATGGACCAATGTGCGTGACTGAC	104
		R: CATGCGGCTCTCCTTCACAACAG	
	SOD1	F:TCTCTCTGACTGGACCACACTGC	100
		R: GTTAGCGTGCTCTCGTTGTCTCC	
	NQO1	F: GCTCTCACGGCACGACATCAC	84
		R: TCCTTCCACGCTTCTCCCATCTC	
	GST	F: GACTTTCTTGTGGGGAACCGACTC	102
		R: AGGAAACCCAGAAAGCACAGCAG	
	GCLM	F: TGTTGTGTGATGCCACCTGATCTC	150
		R: CCATTCGTGTGCTTTGACGTTCTG	
	GCLC	F: TTCAGGTGACATTCCAGGCTTGC	108
		R: AGAACGGAGATGCAGCACTCAATG	
Detoxification	CYP1A1	F: AGGACGGAGGCTGACAAGGTG	104
		R: AGGATGGTGGTGAGGAAGAGGAAG	
	CYP1A4	F: AATGCTCGTTTCAGTGCCTTC	127
		R: CCTCCCCTGTCCTTTTCTCC	
	CYP3A4	F: GACGTGCAGAAGCGACTCCAG	161
		R: TTCTTGCAGATCCGCTCAATCCG	
Inflammation	IL-10	F: CAACCTGCTGCTGAGCCTGAAG	133
		R: CGCCTTGTAGATGCCGTTCTCG	
	IL-16	F: TCAACGGACTCGCAGTAACTCAAC	95
		R: GATCTCTGAAGGGCACGGCTTTC	
	IL-18	F: ACCCTCCATCGCTTCCTTCGG	113
		R: CCGCTGCCAGATTTCATCTCCTG	
	TNF-α	F: CCGTGGTCAGTTTCCATCAGG	117
		R: ACTTTGCAGTTAGGTGACGCT	
	NF-κB	F: GGAGCAGTGGCGGTGTCAAC	126
		R: AGTGCAGTTCATGTCATCGGTCTC	
	Sirt1	F: TGACAGAGCCTCACATGCAAGTTC	144
		R: GGTGGAGGGATTGTTTCTGGTAGC	
Apoptosis	Caspase3	F: TGAGGCAGACAGTGGACCAGATG	110
		R: CTGCATTCCGCCAGGAGTAATAGC	
	Caspase9	F: TGGATTGCGATTCACCCGAAGATG	83
		R: ATTACCCGAGGGAGCCTGGAAAG	
	Bax	F: GCGGACGGAGCCTTCAACTG	84
		R: CTGCGAGAACAGAGCCTTGATGG	
	Bcl-2	F: GAGTTCGGCGGCGTCATGTG	155
		R: CCATACAACTCCACGAAGGCATCC	
	P53	F: ACTGCTACGTCGCGGCTCTC	199
		R: CGCTGGCAAGGCTGGTGAAC	
Internal	β-actin	F: ATGTCGCCCTGGATTTCG	62
reference		R: ATGTCGCCCTGGATTTCG	

Nrf2, nuclear factor erythroid 2-related factor 2; Keap1, Kelch-like ECH-associated protein; HO-1, heme oxygenase 1; CAT, catalase; SOD, superoxide dismutase; NQO1, NAD(P)H quinone oxidoreductase 1; GCLC, glutamate cysteine ligase catalyzes subunits; GCLM, glutamic acid cysteine ligase modified subunit; Caspase-1, cysteine-dependent aspartate-directed protease-1; CYP1A1, cytochrome P450 1A1; CYP1A4, cytochrome P450 1A4; CYP3A4, cytochrome P450 3A4; IL-10, interleukin-10; IL-16, interleukin-16; IL-18, interleukin- 18; TNF-α, tumor necrosis factor α; NF-κB, nuclear factor-κb; Sirt1, Sirtuin 1; Caspase-3, cysteine-dependent aspartate-directed protease-3; Caspase-9, cysteine-dependent aspartate-directed protease-9; Bax, Bcl-2 associated X; Bcl-2, Mus musculus B cell leukemia/lymphoma 2.

**Table 3:** Effects of Res on liver function of duck exposed to AFB1.

Item	Control	AFB1	AFB1 + Res
TP, g/L	35.83 ± 1.62 ^a^	31.17 ± 1.14 ^b^	30.17 ± 0.95 ^b^
AST, IU/L	42.17 ± 9.72	45.20 ± 5.72	42.60 ± 5.45
ALT, IU/L	21.20 ± 0.80 ^b^	34.67 ± 3.04 ^a^	31.25 ± 1.49 ^a^
ALP, IU/L	285.75 ± 11.46	312.00 ± 18.80	304.25 ± 39.19
TBIL, μmol/L	1.43 ± 0.12	1.37 ± 0.049	1.32 ± 0.07
ALB, g/L	17.27 ± 0.60 ^a^	15.83 ± 0.55 ^a,b^	15.43 ± 0.44 ^b^
GLO, g/L	18.57 ± 1.1 ^a^	15.33 ± 0.65 ^b^	14.70 ± 0.64 ^b^
LDH, U/L	1042.24 ± 6.75 ^b^	1219.82 ± 62.32 ^a^	1126.60 ± 34.06 ^a,b^

TP, total protein; ALT, alanine aminotransferase; AST, aspartate aminotransferase; ALP, alkaline phosphate; TBIL, total bilirubin; ALB, albumin; GLO, globulin; LDH, lactate dehydrogenase. Values are represented as the mean ± SEM (*n* = 6). ^a,b^ Mean values with same superscript letters or no letters within a row were of no significant difference (*p* > 0.05), those with different superscript letters were of significant or extremely significant difference (*p* < 0.05).

**Table 4:** Effects of Res on the antioxidative levels of duck liver exposed to AFB1.

Item	Control	AFB1	AFB1 + Res
SOD, U/mg	572.25 ± 16.70 ^a^	382.44 ± 8.52 ^b^	538.71 ± 3.98 ^a^
T-AOC, U/mg	3.82 ± 0.09 ^a^	1.69 ± 0.08 ^c^	2.77 ± 0.13 ^b^
H_2_O_2_, mmol/g	7.50 ± 0.26 ^b^	8.30 ± 0.56 ^a^	7.19 ± 0.2 ^a,b^
CAT, U/mg	31.83 ± 0.49 ^a^	18.35 ± 1.51 ^c^	26.01 ± 0.52 ^b^
MDA, U/mg	1.17 ± 0.12	1.27 ± 0.10	1.29 ± 0.03

SOD, superoxide dismutase; T-AOC, total antioxidant capacity; CAT, catalase; MDA, malondialdehyde; H_2_O_2_, hydrogen peroxide. Values were represented as the mean ± SEM (*n* = 6). ^a–c^ Mean values with same superscript letters or no letters within a row were of no significant difference (*p* > 0.05), those with different superscript letters were of significant or extremely significant difference (*p* < 0.05).

## Data Availability

The raw data presented in this study are available on request from the corresponding author.

## Supplementary Materials

The following are available online at https://www.mdpi.com/article/10.3390/ani11123516/s1, Figures S1: Western Blot Figures.
