# Supplementary material for: Resveratrol Relieved Acute Liver Damage in Ducks (Anas platyrhynchos) Induced by AFB1 via Modulation of Apoptosis and Nrf2 Signaling Pathways
Source: Animals (Basel). 2021 Dec 10;11(12):3516. doi: 10.3390/ani11123516 (PMC8698071; doi:10.3390/ani11123516)
Supplement: Supplementary file 1 [file animals-11-03516-s001.zip › Figure S1 Western Blot Figures.pdf]

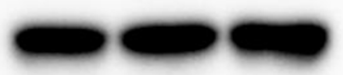
GAPDH
CON AFB1 AFB1+Res

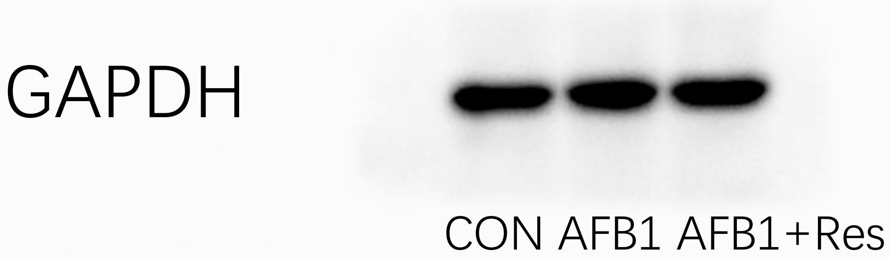
GAPDH
CON AFB1 AFB1+Res

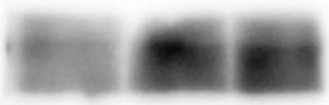
CYP1A1
CON AFB1 AFB1+Res

CYP3A4

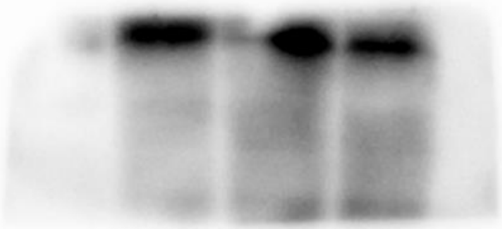

CON AFB1 AFB1+Res

NF-kb/P65

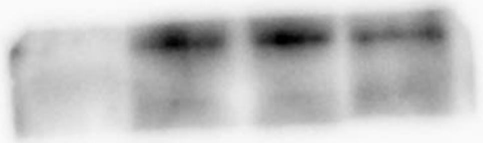

CON AFB1 AFB1+Res

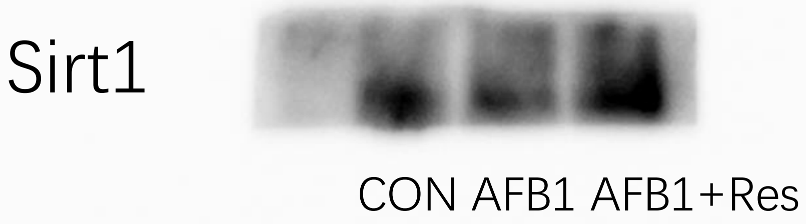
Sirt1
CON AFB1 AFB1+Res

HO-1

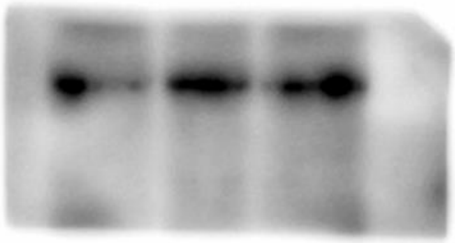

CON AFB1 AFB1+Res

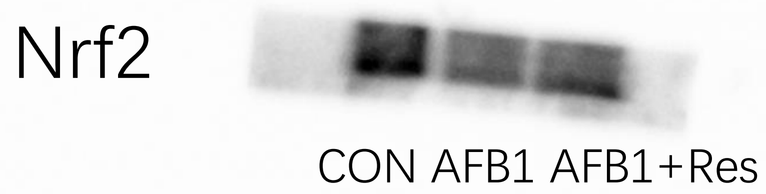
Nrf2
CON AFB1 AFB1+Res

Keap1

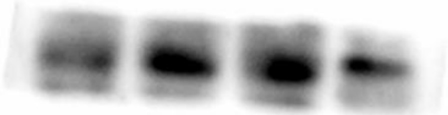

CON AFB1 AFB1+Res

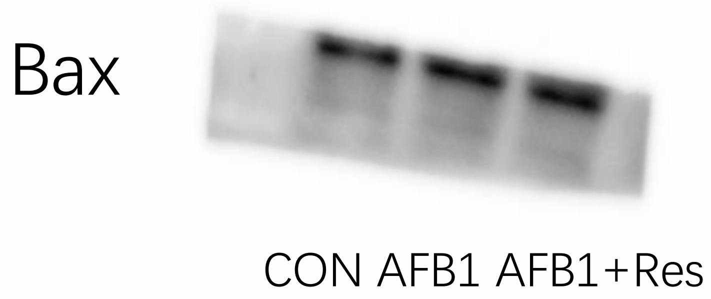
Bax
CON AFB1 AFB1+Res

Cyt-c

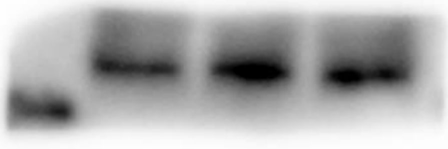

CON AFB1 AFB1+Res

Cleaved
Caspase9

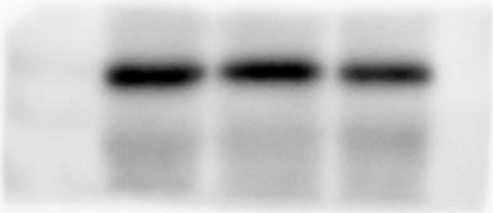

CON AFB1 AFB1+Res

Cleaved
Caspase3

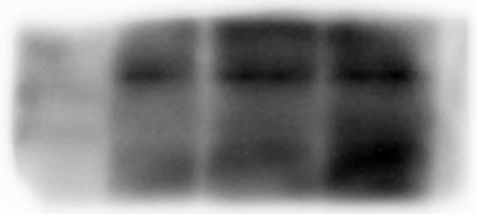

CON AFB1 AFB1+Res

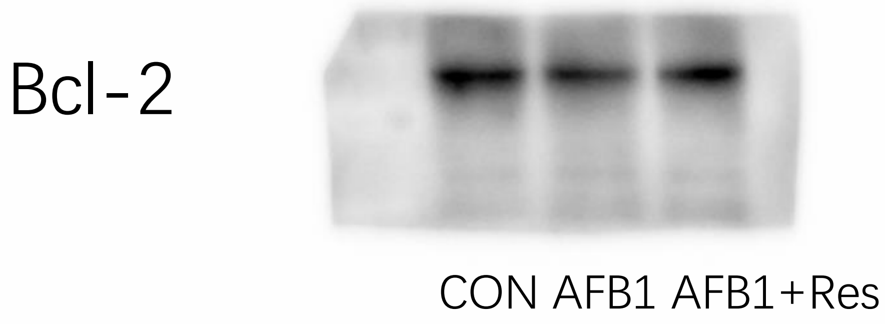
Bcl-2
CON AFB1 AFB1+Res
